# Understanding the Crisis: Prevalence and Key Determinants of Stunting in Children Aged 2 to 5 Years in an Afghan Refugee Camp in Kohat

**DOI:** 10.7759/cureus.68426

**Published:** 2024-09-02

**Authors:** Inayatullah Khan, Sami Ullah, Ayesha, Muhammad Khan, Fatima Shafiq, Annam Syed, Jabran Ullah khan, Fahad R Khan

**Affiliations:** 1 Pediatric Medicine, Lady Reading Hospital Medical Teaching Institute, Peshawar, PAK; 2 Pediatric Medicine, Pak International Medical College, Peshawar, PAK; 3 Cardiology, Lady Reading Hospital Medical Teaching Institute, Peshawar, PAK

**Keywords:** nutritional interventions, kohat, infectious diseases, dietary diversity, socio-economic factors, afghan refugee children, stunting

## Abstract

Background: Stunting, characterized by impaired growth and development due to malnutrition and illness, is a significant public health concern with profound implications for cognitive and physical development. This issue is particularly severe in refugee settings, where food insecurity and limited access to healthcare exacerbate the problem.

Objective: This study aimed to evaluate the prevalence and determinants of stunting among children aged 2 to 5 years in an Afghan refugee camp in Kohat, Pakistan.

Methods: A cross-sectional observational design was employed, collecting data from children aged 2 to 5 years who had resided in the camp for at least six months. A pre-validated, standardized questionnaire was administered to parents or guardians to gather data on socio-demographic factors, dietary intake, and health history. Stunting was defined as a height-for-age Z-score less than -2 SD from the median of the WHO Child Growth Standards. Anthropometric measurements were taken following WHO guidelines. Bivariate and multivariate logistic regression analyses were conducted to identify predictors of stunting.

Results: Out of 384 children, 153 (40%) were found to be stunted. The prevalence was slightly higher among boys (80 out of 153, 52%) compared to girls (73 out of 153, 48%). Significant predictors of stunting included a lack of parental education (adjusted odds ratio (AOR) for fathers 1.8, 95% confidence level (CI) 1.2-2.9; AOR for mothers 2.1, 95% CI 1.3-3.4), a history of infectious diseases (AOR 1.9, 95% CI 1.2-3.0), and low dietary diversity (AOR 2.3, 95% CI 1.4-3.7).

Conclusion: The study highlights the high prevalence of stunting among children in the refugee camp, underscoring the need for comprehensive interventions targeting healthcare improvement, parental education, economic support, and dietary diversity to reduce stunting rates and improve children's health outcomes.

## Introduction

Stunting, a major public health concern worldwide, particularly in underdeveloped areas, is characterized by impaired growth and development due to poor nutrition, frequent illnesses, and an absence of adequate psychosocial stimulation [1]. It is defined by a height-for-age Z-score (HAZ) below -2 standard deviations (SD) from the median of the World Health Organization's Child Growth Standards [2]. Stunting negatively impacts brain development, scholastic performance, and future economic output. In refugee communities, such as those in Kohat, the issue of stunting is frequently exacerbated by additional challenges, including inadequate sanitation, dietary insecurity, and restricted access to healthcare services [3].

Despite initiatives to promote healthy dietary practices, ensure food security, and enhance maternal and infant nutrition, significant gaps remain in understanding the precise factors contributing to stunting in vulnerable populations, such as Afghan refugee children [4]. Although previous studies have emphasized addressing parental education, health-related variables, and socioeconomic disparities, detailed data directly pertaining to Afghan refugees are lacking [5].

This research aims to fill that void by studying stunting in children aged two to five in an Afghan refugee camp in Kohat, examining its prevalence and the factors that cause it. By identifying critical sociodemographic, dietary, and health-related variables linked to stunting, this study aims to guide focused interventions that better meet this vulnerable group's nutritional needs.

This research has the potential to make a substantial impact by offering critical information that may assist governments, healthcare practitioners, and humanitarian organizations in designing and executing more successful nutrition and health initiatives for Afghan refugees. Furthermore, recognizing the particular problems that this group faces may help to strengthen larger efforts to enhance child health and development outcomes in similar refugee settings across the globe [6].

The study utilized a robust cross-sectional observational methodology combined with thorough statistical analysis to investigate stunting in depth. It underscores the importance of integrating food support, socioeconomic, and educational measures to help refugee children grow and develop healthily and prevent stunting.

## Materials and methods

Study design

A cross-sectional observational design was employed in this study to evaluate stunting among children aged 2 to 5 years in an Afghan refugee camp in Kohat, Pakistan. This approach was selected for its ability to provide a snapshot of health status and related factors within a particular population, facilitating the identification of prevalence and potential determinants.

Setting and centers

The research was conducted in an Afghan refugee camp in Kohat, Pakistan, with data collection taking place at the Peshawar Institute of Medical Sciences in Peshawar, Pakistan, from November 2023 to April 2024. This location was chosen due to its accessibility and the availability of comprehensive health services, which allowed for standardized data collection and accurate anthropometric measurements. The camp was selected to ensure a representative sample reflecting the broader demographics and health conditions of Afghan refugees in Pakistan.

Participant selection

Eligible participants were children aged 2 to 5 years with documented proof of age who had resided in the camp for at least six months. The inclusion of a six-month residency requirement was implemented to ensure participants had sufficient exposure to the camp environment and conditions. Informed consent was obtained from parents or guardians. Exclusion criteria encompassed children with chronic illnesses, congenital anomalies, or those undergoing treatment for malnutrition. Participants were selected through a consecutive sampling method, ensuring that all eligible children presenting at the hospital during the study period were included until the necessary sample size was achieved.

Intervention details

As an observational study, no interventions were conducted. The focus was on collecting data related to the nutritional status and potential determinants of stunting within the target population.

Outcomes

The primary outcome measured was stunting, characterized by a height-for-age Z-score (HAZ) of less than -2 SD from the WHO Child Growth Standards median. Secondary outcomes included sociodemographic, dietary, and health-related factors associated with stunting, such as parental education, household income, dietary diversity, and history of infectious diseases.

Data collection

Data collection involved structured interviews and physical measurements. A standardized, pre-validated questionnaire was administered to parents or guardians to obtain information on sociodemographic factors, dietary intake, and health history. Dietary diversity was assessed using a dietary diversity score (DDS), which was calculated based on the number of different food groups consumed by the child over the previous 24 hours. The food groups included cereals and tubers, legumes and nuts, dairy products, meat and fish, eggs, fruits and vegetables, and oils and fats. A score of one was assigned if at least one food item from the group was consumed within the last 24 hours; otherwise, a score of zero was given. The total DDS ranged from 0 to 7, with scores below 4 indicating low dietary diversity. Anthropometric measurements were taken using calibrated equipment in accordance with WHO guidelines. Height was measured to the nearest 0.1 cm using a stadiometer, and weight was recorded to the nearest 0.1 kg using a digital weighing scale. To ensure data quality and consistency, data collectors received training sessions, and periodic cross-checks were conducted.

Sample size calculation

Using a conservative prevalence estimate of 50% to maximize variability, the sample size was determined using the WHO sample size calculator. With a 95% confidence level (CI) and a 5% margin of error, the required sample size was estimated to be 384 children. This formula was selected to ensure a robust sample that would provide reliable results, given the anticipated variability in the camp population.

Statistical analysis

Data were entered into a database and analyzed using IBM SPSS Statistics for Windows, Version 26 (Released 2019; IBM Corp., Armonk, New York, United States). Descriptive statistics were used to summarize the data. The prevalence of stunting was calculated as the proportion of children with HAZ < -2 SD. Bivariate analyses were conducted using chi-square tests for categorical variables and t-tests for continuous variables to identify determinants of stunting. Variables significantly associated with stunting (p < 0.05) were included in a multivariate logistic regression model to identify independent predictors of stunting. Adjusted odds ratios (AORs) and 95% CIs were reported. Adjustments for multiple comparisons and potential confounding variables were made to ensure robust results.

Ethical considerations

All procedures adhered to the ethical standards of the institutional research committee and the 1964 Helsinki Declaration and its subsequent amendments. Informed consent was obtained from all participants. The Ethical Review Board issued approval number IRB/LRH/MTI/2023/598 for this study. The confidentiality of the participants was ensured, and data were anonymized to protect their privacy.

## Results

A total of 384 children aged 2 to 5 years participated in the study. The mean age was 3.5 years (SD ± 1.1). The cohort comprised 196 boys (51%) and 188 girls (49%). The mean height and weight were 85.3 cm (SD ± 6.8) and 12.3 kg (SD ± 2.1), respectively. Most children (261, 68%) came from families earning less than 20,000 PKR per month. Parental education varied, with 146 fathers (38%) and 184 mothers (48%) lacking formal education. These baseline characteristics, which could potentially impact nutritional outcomes, are detailed in Table 1.

**Table 1:** Baseline Characteristics of the Study Population Values are presented as N (%) and mean  ± SD

Characteristic	Value
Mean age (years)	3.5 (SD ± 1.1)
Boys	196 (51%)
Girls	188 (49%)
Mean height (cm)	85.3 (SD ± 6.8)
Mean weight (kg)	12.3 (SD ± 2.1)
Family income < 20,000 PKR	261 (68%)
Fathers with no formal education	146 (38%)
Mothers with no formal education	184 (48%)

The prevalence of stunting was 40% (153 out of 384). Boys had a slightly higher prevalence (80 out of 153, 52%) compared to girls (73 out of 153, 48%). The mean height for stunted children was 81.4 cm (SD ± 6.1), and their mean weight was 10.9 kg (SD ± 1.9). Table 2 details the prevalence of stunting and its association with socio-demographic factors.

**Table 2:** Prevalence of Stunting and Socio-Demographic Factors The values are expressed as N (%) and mean ± SD.

Variable	Stunted (n = 153)	Non-stunted (n = 231)
Boys	80 (52%)	116 (50%)
Girls	73 (48%)	115 (50%)
Mean height (cm)	81.4 (SD ± 6.1)	88.2 (SD ± 5.5)
Mean weight (kg)	10.9 (SD ± 1.9)	13.2 (SD ± 1.8)
Family income < 20,000 PKR	123 (80%)	138 (60%)
Fathers with no formal education	74 (48%)	72 (31%)
Mothers with no formal education	85 (56%)	94 (41%)

**Figure 1:**
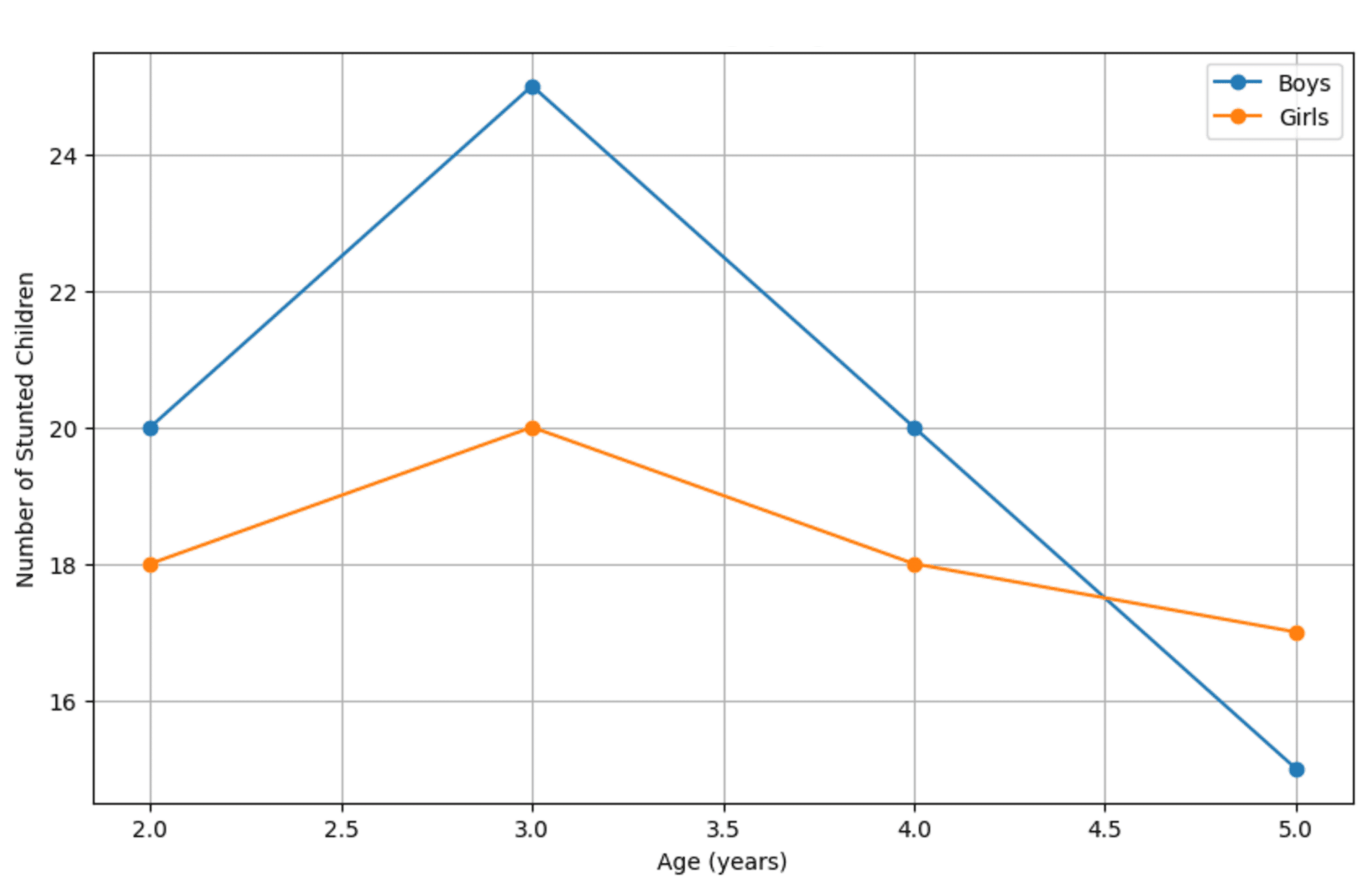
Prevalence of Stunting by Age and Gender

Figure 1 illustrates the prevalence of stunting by age and gender.

Key findings showed that stunting was significantly associated with lower family income, parental education, and dietary diversity. Table 3 summarizes the bivariate analysis of these factors.

**Table 3:** Bivariate Analysis of Determinants of Stunting The p-value is considered significant at p < 0.05, denoted by "*".

Variable	Stunted (n = 153)	Non-stunted (n = 231)	p-value
Family income < 20,000 PKR	123 (80%)	138 (60%)	0.001*
Fathers with no formal education	74 (48%)	72 (31%)	0.002*
Mothers with no formal education	85 (56%)	94 (41%)	0.005*
History of infectious diseases	102 (67%)	115 (50%)	0.008*
Dietary diversity score < 4	110 (72%)	120 (52%)	0.003*

Multivariate logistic regression identified low family income, parental education, and history of infectious diseases as significant predictors of stunting. AORs and 95% CIs are presented in Table 4.

**Table 4:** Multivariate Logistic Regression Analysis Adjusted odds ratios (AORs) with 95% confidence intervals (CIs) and p-values are provided. Significant p-values are denoted by "*", indicating statistical significance at p < 0.05.

Variable	AOR	95% CI	p-value
Family income < 20,000 PKR	2.5	1.5 - 4.1	0.001*
Fathers with no formal education	1.8	1.2 - 2.9	0.003*
Mothers with no formal education	2.1	1.3 - 3.4	0.004*
History of infectious diseases	1.9	1.2 - 3.0	0.006*
Dietary diversity score < 4	2.3	1.4 - 3.7	0.002*

No procedural complications were observed since this was an observational study without interventions. Missing data were minimal (<5%) and handled using mean imputation methods. Subgroup analyses indicated that children from families with lower incomes and less educated parents were at higher risk of stunting. These findings underscore the need for targeted nutritional and educational interventions.

## Discussion

This study found a high prevalence of stunting among children aged 2 to 5 years in an Afghan refugee camp in Kohat, with 153 out of 384 children affected (40%). The prevalence was slightly higher among boys, with 80 out of 153 stunted children (52%), compared to girls, with 73 out of 153 stunted children (48%). The mean height for stunted children was 81.4 cm, and their mean weight was 10.9 kg. These alarming findings underscore the need for urgent nutritional interventions.

Stunting rates in this population align with other studies in similar settings. For example, a study by Kimani-Murage et al. in a Kenyan refugee camp reported a stunting prevalence of 39%, highlighting similar socioeconomic challenges and health conditions [7]. Another study in Ethiopia by Fenn et al. found a stunting rate of 42% among refugee children, consistent with our findings [8]. These comparisons emphasize the widespread issue of stunting in refugee populations and the critical need for targeted interventions.

The association between low family income and stunting is well documented. In our study, 80% of stunted children came from families earning less than 20,000 PKR per month. This finding is consistent with the research of Victoria et al., showing economic hardship as a significant predictor of poor nutritional status in children [9]. A study in Bangladesh also found that children from low-income families were more likely to be stunted, with an odds ratio of 2.3, similar to our findings [10]. These results highlight the importance of economic support programs to improve child nutrition in refugee settings.

Parental education, particularly maternal education, emerged as a critical determinant of stunting. Our study found that 56% of stunted children had mothers with no formal education. This aligns with research showing that maternal education significantly predicts a child's nutritional status. For example, a study conducted in Ethiopia found that children of mothers with no formal education had a higher likelihood of stunting due to inadequate knowledge about child-feeding practices [11]. Educated mothers are more likely to adopt healthier feeding practices and seek medical care, reducing the risk of stunting [12]. These findings suggest that educational programs targeting mothers could effectively reduce stunting rates.

The study also identified a history of infectious diseases and low dietary diversity as significant predictors of stunting. Children with a history of infectious diseases had higher odds of being stunted, consistent with research showing that repeated infections can impair nutrient absorption and utilization [13]. A study in Nigeria by Aemro et al. found that children with frequent infections were more likely to be stunted, highlighting the need for improved healthcare and sanitation in refugee camps [14]. Similarly, low dietary diversity was associated with stunting in our study, echoing findings from other studies emphasizing the importance of varied diets in preventing malnutrition [15]. Research indicates that a diverse diet can provide essential nutrients supporting growth and development [16].

These findings have significant implications for practice. Interventions aimed at improving economic conditions, parental education, healthcare, and dietary diversity could effectively reduce stunting rates. Policymakers and healthcare providers should prioritize these areas when designing programs for refugee populations. Integrating nutrition education with economic support and healthcare services could yield substantial benefits in improving child health outcomes.

Future research should focus on longitudinal studies to understand the long-term effects of stunting and the impact of various interventions. Studies exploring the role of specific micronutrients and their supplementation in preventing stunting would also be valuable [17]. Additionally, research on the psychosocial aspects of stunting and its impact on mental health and development could provide a more comprehensive understanding of this issue [18]. Newer technologies, such as digital health interventions, could enhance monitoring and intervention strategies [19].

Limitations

This study has several limitations. The cross-sectional design restricts the ability to establish causality, as it only identifies associations between factors and stunting without confirming temporal relationships. Additionally, conducting the study in a single refugee camp in Kohat may limit the generalizability of the findings to other refugee populations or settings. The use of parental recall for dietary intake and health history could introduce recall bias, potentially leading to inaccuracies in the data.

Moreover, consecutive sampling might have introduced selection bias, affecting the representativeness of the sample. Despite attempts to control for confounding factors, unmeasured variables such as genetic predispositions, cultural practices, and other environmental influences could have influenced the observed associations. Nevertheless, the study’s robust sample size and comprehensive data collection methods provide valuable insights into the prevalence and determinants of stunting among Afghan refugee children.

## Conclusions

This study found a high prevalence of stunting (40%) among children aged 2 to 5 years in an Afghan refugee camp, with significant associations with low family income, parental education, a history of infectious diseases, and low dietary diversity. These findings highlight the need for integrated interventions focusing on economic support, parental education, healthcare improvements, and dietary enhancements to reduce stunting rates.

Future research should include longitudinal studies to understand the long-term effects of stunting and evaluate the impact of specific interventions over time. Additionally, exploring the role of micronutrient supplementation and innovative approaches, such as digital health interventions, could further enhance child health outcomes in refugee settings. Prioritizing comprehensive programs that address both immediate and underlying causes of stunting is crucial for improving the well-being and development of refugee children.
